# Anion Specific Effects at Negatively Charged Interfaces: Influence of Cl^–^, Br^–^, I^–^, and SCN^–^ on the Interactions of Na^+^ with the Carboxylic Acid Moiety

**DOI:** 10.1021/acs.jpcb.1c07758

**Published:** 2021-10-27

**Authors:** Adrien P. A. Sthoer, Eric C. Tyrode

**Affiliations:** Department of Chemistry, KTH, Dröttning Kristinas väg 51, SE-10044 Stockholm, Sweden

## Abstract

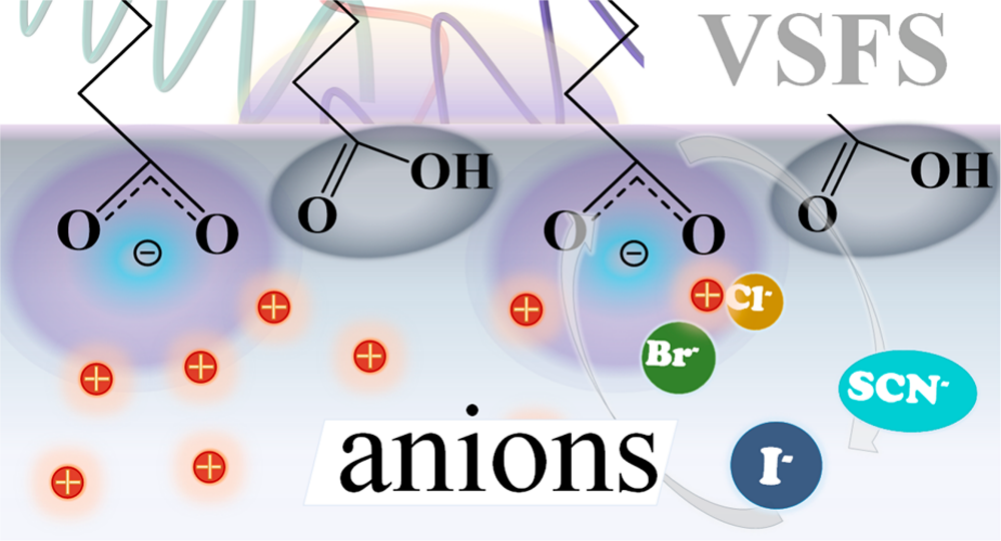

Unlike counterion interactions with charged interfaces, the influence of co-ions is only scarcely reported in the literature. In this work, the effect of SCN^–^ and the halide co-ions in the interactions of Na^+^ with carboxylic acid Langmuir monolayers is investigated by using vibrational sum frequency spectroscopy. At 1 M concentrations in the subphase, the identity of the anion is shown to have a remarkable influence on the charging behavior and degree of deprotonation of the monolayer, with ions ordering in the sequence I^–^ > SCN^–^ > Cl^–^ ≈ Br^–^. The same trend is observed at both pH 6 and pH 9 when the monolayer is intrinsically more charged. Spectroscopic evidence is found for both the presence of I^–^ and SCN^–^ in the interfacial region at levels close to their detection limits. The results contradict electrostatic theories on charged interfaces where co-ions are not expected to play any significant role. The higher propensity for the large polarizable anions to deprotonate the monolayer is explained in terms of their ability to modify the cations affinity toward the carboxylic acid groups present at the surface.

## Introduction

Fatty acid Langmuir monolayers deposited on aqueous electrolyte solutions are known to acquire a negative charge due to the deprotonation of the carboxylic acid moiety. The ensuing negative surface potential creates an electrical double layer (EDL), where ions in solution redistribute to screen the surface charge.^1,2^ Because of basic electrostatic arguments, counterions (i.e., cations) are enriched at the surface, while co-ions (i.e., anions) are instead expected to be largely expelled. It becomes then obvious why the vast majority of the research on specific interactions of ions with carboxylic acids monolayers has focused on the cations.^3−13^

Anion specific effects are, however, typically more pronounced than those for cations when interacting with hydrophobic or neutral polar surfaces.^14,15^ For instance, in contrast to cations, large polarizable anions have a higher propensity to adsorb to hydrophobic surfaces^16−21^ or to hydrophobic patches in macromolecules.^15,22,23^ Yet, at negatively charged interfaces that do not expose hydrophobic moieties to solution, anions have an additional penalty for adsorption and do not directly interact.^24,25^

Nonetheless, anion specific effects have been previously reported at the negatively charged silica/water interface by using second harmonic generation, where it was observed that in concentrated sodium and potassium halide solutions (i.e., 0.5 M) the apparent p*K*_a_ of the surface silanol groups varied depending on the identity of the anion.^26^ Though the surface charge could not be experimentally measured, it was proposed that the more polarizable halides promoted a larger surface deprotonation.^26^ Subsequent experiments on silica particles where the surface charge density was directly measured using potentiometric titration, FTIR, and X-ray photoelectron spectroscopy concluded, albeit for lower salt concentrations (i.e., 50 mM), that the identity of the anion had actually no significant effect.^27^ However, later experiments using a similar experimental approach showed that anion effects are indeed apparent when the ion concentration is increased beyond 0.2 M.^28^ In connection with these studies, the strong dependence on the co-ion identity and concentration of pH values measured with glass electrodes has also been ascribed to anion specific interactions at the negatively charged glass-electrode surface.^29,30^ Though more subtle, halide anions have been shown to have an influence on the interfacial water structure at negatively charged alumina surfaces.^31^ Anion specific effects have also been reported on a negatively charged dihexadecyl phosphate monolayer, but only in the presence of trivalent cations in solution.^32^ In such a case, the preferential adsorption of the trivalent cation leads to charge reversal and a net positively charged surface,^8^ where anions can more readily interact. No such effect is expected for monovalent cations.

In the specific case of carboxylic acid monolayers, only one experimental study has focused on the effect of the co-ions at low ionic strengths (i.e., 1 mM), where it was concluded, in agreement with the Gouy–Chapman theory, that the identity of the anion (or monovalent cation for that matter) had no effect on the observed charging behavior.^7^ Moreover, MD simulations predict that the Cl^–^ and I^–^ are equally depleted for the carboxylate headgroups but that chloride has a slightly higher preference to interact with the uncharged carboxylic acid moiety.^33,34^

In this study, we use vibrational sum frequency spectroscopy (VSFS) to investigate the potential effects the identity of the co-ion can have in the charging behavior of carboxylic acid monolayers deposited on concentrated salt solutions having sodium as the common counterion. From the analysis of the headgroup vibrations, the degree of deprotonation and the type of ion pairs formed can be extracted. The results show a striking dependence on the anion identity. VSFS also provides additional information about the interfacial water molecules as well as direct and indirect evidence for the presence of thiocyanide and iodide anions in the interfacial region.

## Methods

### Materials

NaCl (99.999% trace metals basis), NaI (99.999%, trace metals basis), NaBr (99.99%, trace metals basis), NaSCN (99.99%, trace metals basis), NaOH (99.99%, trace metals basis), Na_2_S_2_O_3_ (99.99%, trace metal basis), ethylenediaminetetraacetic acid (EDTA, 99.995%, trace metals basis), eicosanoic-*d*_39_ acid (97%, dAA for deuterated arachidic acid), eicosanoic acid (99%, AA for arachidic acid), and chloroform (anhydrous grade, stabilized with ethanol) were obtained from Merck. HCl, 36.5% (99.999%, trace metal basis), was purchased from Alfa-Aesar. Before use, NaCl, NaBr, and NaI were baked at 500 °C for 1 h and slowly cooled to eliminate any traces of organic compounds. As NaSCN decomposes at a significantly lower temperature, the salt was cleaned using an approach similar to that proposed by Lunkenheimer for purifying surfactants,^35−37^ where the surface of NaSCN stock solutions are repeatedly aspirated to remove traces of surface active contaminants. The remaining compounds were used as received. Ultrapure water was obtained from an Integral 15 Millipore system featuring a constant conductivity (18.2 MΩ·cm) and low total organic content (<3 ppb). The glassware was cleaned beforehand by a three-step sonication procedure with, subsequently, ethanol, Deconex (Borer Chemie), and ultrapure water, alternated with 10 times rinsing with water between each step.

### Solution Preparation

All solutions were prepared in a background of 20 μM EDTA, for which the pH had been adjusted before adding the salt. The addition of EDTA is critical for avoiding trace amounts of divalent/trivalent ions from interacting with the fatty acid monolayer.^6,8^ Anion specific interactions were observed at the silica surface of the pH electrode, particularly for the more polarizable anions such as SCN^–^ and I^–^. Thus, the pH was systematically verified before and after experiments with different pH indicator papers (MColorpHast pH 0–14, 4.0–7.0, 6.5–10, and 11.0–13.0 and Whatman Panpeha pH 0–14 from Merck). As elaborated in the Supporting Information, iodide can oxidize in contact with oxygen at ambient conditions, a reaction that is catalyzed by light. To prevent or retard the reaction kinetics, NaI solutions were freshly prepared ∼1 to 2 h before each experiment and kept in a dark environment. Moreover, to confirm that our results did not originate from trace amounts of iodate anions that may consume H^+^ ions changing the pH, solutions with 50 mM of the strong reducing agent Na_2_S_2_O_3_ were also tested, showing no measurable differences (see the Supporting Information for a detailed description of the iodide chemistry). The AA/dAA solutions for the trough deposition were prepared by diluting 10 mg of fatty acid in ∼10 mL of chloroform.

### Fatty Acid Langmuir Monolayers

The monolayer is formed by spreading ∼10 μL of the amphiphile solution on the electrolyte solution placed in a Langmuir trough from KSV NIMA (195 mm length, 50 mm width, and 4 mm in depth). Before compression, a waiting time of ∼10 min was observed to ensure the full evaporation of the chloroform solvent. The monolayer is compressed with Delrin moving barriers at a rate of 5 mm/min. The surface pressure was measured by using a 10 mm wide paper Wilhelmy plate. All VSF experiments are performed at a constant surface pressure of 20 mN/m and a constant temperature of 22.0 ± 0.5 °C.

### VSF Spectrometer

The femtosecond VSF spectrometer has been described in detail elsewhere.^38^ Briefly, a 1 kHz narrow picosecond pulse centered at 805 nm and a tunable ∼100 fs IR pulse are directed to the sample position in a copropagating geometry, with angles of incidence set to 70° and 55°, respectively. The generated SF signals are collected using an optical setup that displays a high degree of automation, allowing measurements in broad spectral regions. The SF signal is finally detected by using a spectrometer (Shamrock SR202i-B, Andor, Ireland) and an EM-CCD camera (Newton, Andor, Ireland). The powers at the sample position for the IR and “visible” beams were typically set to ∼5 and ∼30 mW, respectively. Spectra were recorded in the polarization combinations SSP, SPS, and PPP, with a spectral resolution <3 cm^–1^, and normalized by the nonresonant SF response from a gold surface.^38^ The VSF spectra were fitted using eq 1, which is a convolution of Lorentzian and Gaussian line shapes that account for the homogeneous and inhomogeneous broadening as well as complex interferences between neighboring bands.^7,39^
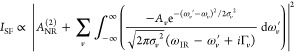
1where *A*_NR_ refers to the nonresonant contribution to the SF signal, *A*_*v*_ to the amplitude or oscillator strength of the *v*th resonant mode, ω_IR_ to the infrared frequency, ω_*v*_ to the peak position, and Γ_*v*_ and σ_*v*_ to the Lorentzian and Gaussian line widths, respectively.

## Results

**Figure 1:**
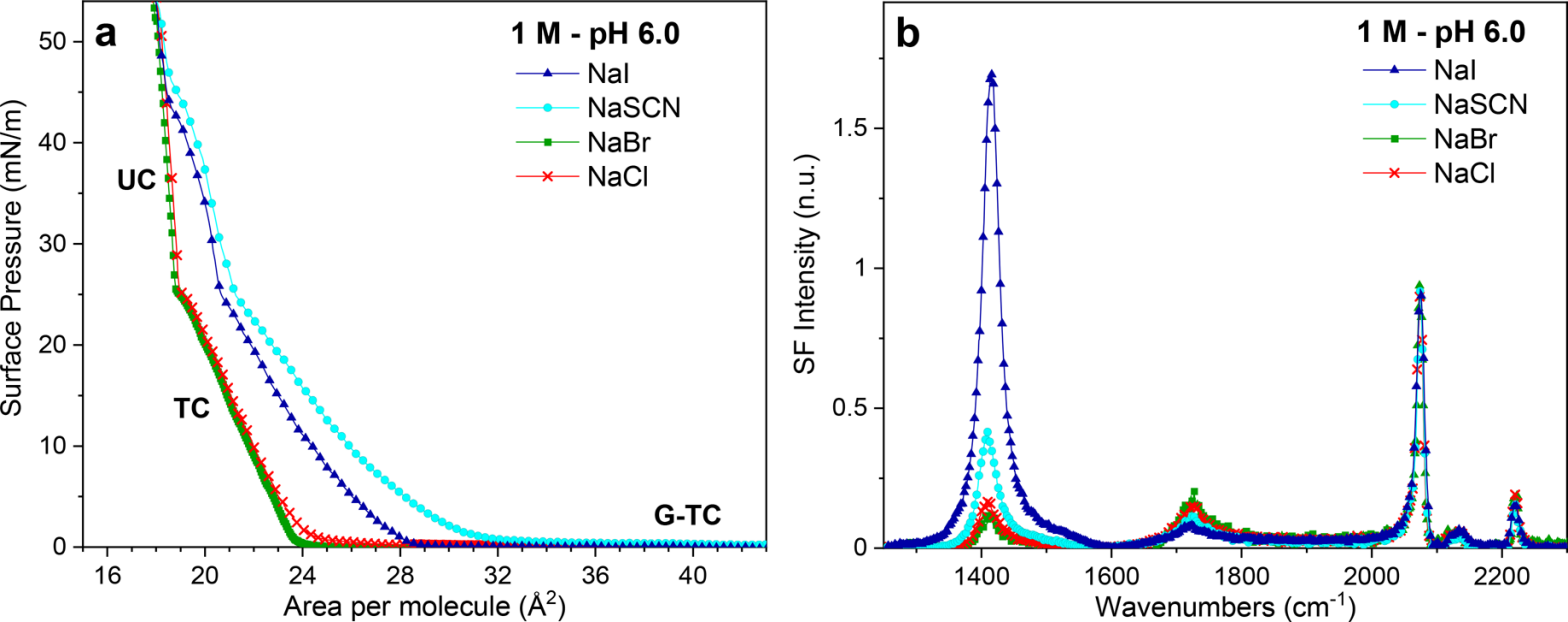
(a) Π–*A* isotherms of arachidic acid monolayers on 1 M solutions of NaCl, NaBr, NaSCN, and NaI at pH 6.0 and 22 °C (G: 2D gas phase; TC: tilted condensed phase; UC: untilted condensed phase). (b) Vibrational sum frequency spectra collected under the SSP polarization of a dAA monolayer on 1 M solutions of NaCl, NaBr, NaSCN, and NaI at pH 6 and constant surface pressure of 20 mN/m (22 °C and 20 μM EDTA).

The surface pressure versus molecular area isotherms (Π–*A*) of arachidic acid monolayers on aqueous subphases of 1 M NaCl, NaBr, NaI, and NaSCN at pH 6 are shown in Figure 1a. The isotherms for NaCl and NaBr are similar to that on a pure water subphase,^6,7,40^ where three distinct regions can be observed. These are the 2D gas (G)–tilted condensed (TC) coexistence region, the pure TC phase, and for the lowest areas per molecule, following the kink in the isotherms at ∼27 mN/m, the untilted condensed (UC) phase (see Figure 1a). Interestingly, the isotherms for NaI and NaSCN are significantly different, showing an evident expansion, which suggests additional repulsive forces within the monolayer. A molecular insight into the underlying causes of these discrepancies is obtained using VSFS.

The SSP-polarized VSF spectra of equivalent monolayers at a constant surface pressure of 20 mN/m are shown in Figure 1b. The spectral range presented focuses on vibrations from the carboxylic acid headgroup (1300–1800 cm^–1^) and the fatty acid’s deuterated alkyl chain (2050–2250 cm^–1^). In the CD stretching range, the sharp bands observed at ∼2075, ∼2135, and ∼2218 cm^–1^ are all linked to the terminal methyl group and assigned respectively to the symmetric (r^+^), Fermi resonance (r^+^_FR_), and asymmetric (r^–^) CD_3_ stretch.^7,41^ The lack of features from the methylene groups indicates that the deuterated alkyl chains are densely packed in an *all*-*trans* configuration. This reflects the high packing densities expected from the Π–*A* isotherms. In this spectral region, no major differences are observed between the different salts, as all monolayers are found in the TC phase and have, within error (±1 Å^2^), similar surface densities.

The vibrational features associated with the carboxylic acid headgroup show, however, obvious differences (see Figure 1b). The main peaks are the symmetric carboxylate stretch at ∼1408 cm^–1^ (*v*_*s* COO_^–^) and the carbonyl stretch (*v*_C=O_) at ∼1720 cm^–1^, the presence of which indicates that for all salt solutions the monolayers are only partly deprotonated. Nonetheless, the relative intensities significantly vary depending on the identity of the anion. The spectrum on NaI displays the most intense carboxylate stretch, followed by NaSCN and finally NaCl and NaBr, which behave similarly. The *v*_C=O_ linked to the uncharged carboxylic acid groups in the monolayer shows an opposite trend, confirming that the percentage of deprotonation depends on the identity of the co-ion in the order I^–^ > SCN^–^ > Cl^–^ ≈ Br^–^. Strikingly, the differences between the anions in sodium salts are substantially larger than those observed when varying the identity of the monovalent cation in chloride salts at the same pH.^6^ A closer inspection of the data at pH 6 shows that the *v*_*s* COO_^–^ band is centered at ∼1408 cm^–1^ for NaCl, NaBr, and NaSCN but slightly blue-shifted and broader for NaI. The former peak position has been associated with hydrated carboxylate species,^3,7,42^ while the blue-shifted peak is indicative of closer interactions between the cation and the carboxylate, as, for example, the formation of contact ion pairs (CIP).^5,6^ This effect will be further discussed below when quantifying the degree of deprotonation of the monolayer.

**Figure 2:**
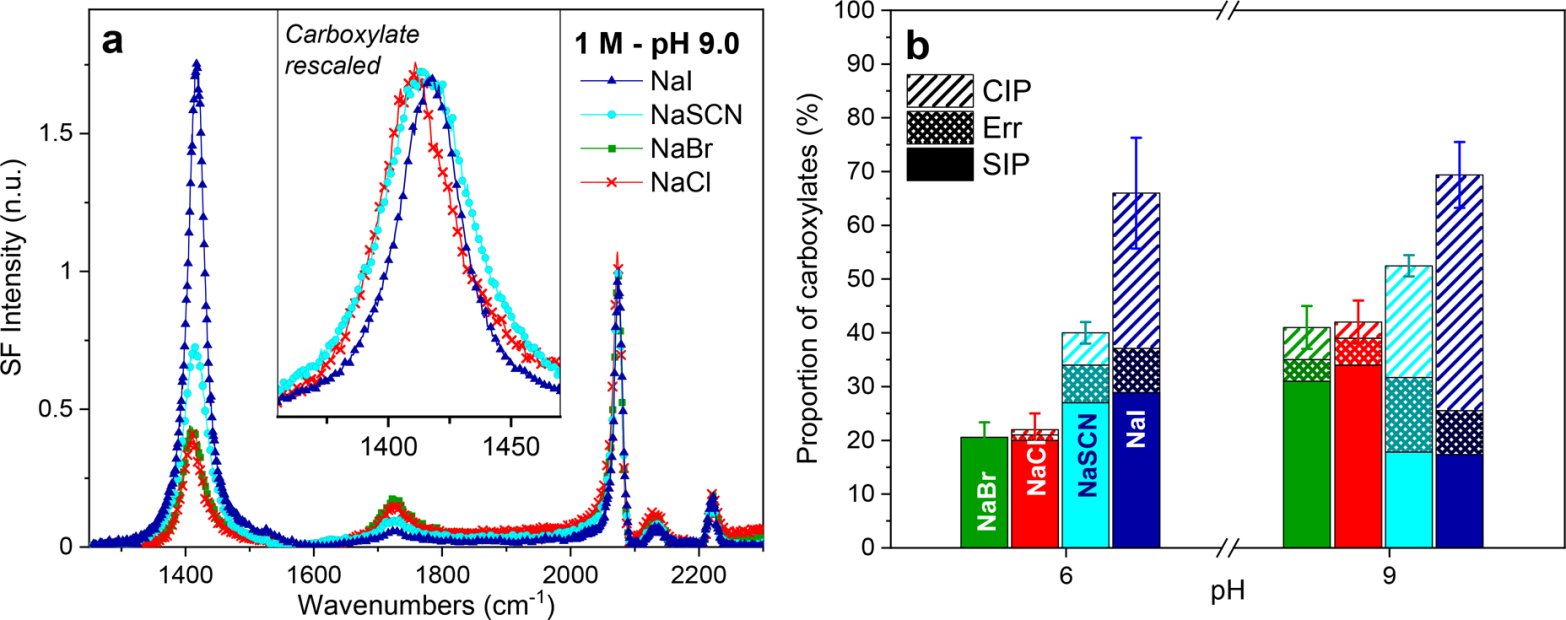
(a) Vibrational sum frequency spectra collected under the SSP polarization of a deuterated arachidic acid monolayer on 1 M solutions of NaCl, NaBr, NaSCN, and NaI at pH 9 and a constant surface pressure of 20 mN/m (22 °C and 20 μM EDTA). The inset highlights the blue-shift observed for the symmetric carboxylate stretch linked to the formation of a contact ion pair (CIP) for the different salts. (b) Percentage of monolayer deprotonation and relative contributions of the hydrated carboxylate (SIP) and CIP, for NaCl (red), NaBr (green), NaSCN (cyan), and NaI (blue) at pH 6.0 and 9.0. The relative error (Err) in the carboxylate proportions is also plotted for each salt. The error bars result from the fits of multiple repeat experiments (see the Supporting Information for additional details).

To investigate the influence of the intrinsic surface charge in the anion specificity, experiments were also performed at a higher pH. The VSF spectra of dAA monolayers on 1 M NaCl, NaBr, NaI, and NaSCN at pH 9 are shown in Figure 2a. When only the pH determining ions (e.g., OH^–^) are present in solution, the arachidic acid monolayer has been shown to be essentially uncharged (<0.5%) at pH ∼ 6, while at pH 9 it is ∼18% deprotonated.^7^ Similarly, in the presence of 1 M salt in the subphase, the dAA monolayers are also more deprotonated at pH 9. This is deducted from the relatively higher intensities in the carboxylate stretching region for the corresponding salts (Figure 2a compared with Figure 1b). Yet, more importantly, the monolayer deprotonation shows the same dependency with the anion identity as pH 6, with I^–^ > SCN^–^ > Cl^–^ ≈ Br^–^. Moreover, as the monolayers are more deprotonated, the shift in frequency of *v*_*s* COO_^–^ is more apparent, as shown in the inset of Figure 2a. The band is centered at ∼1417 cm^–1^ for NaI and ∼1408 cm^–1^ for NaCl with a shoulder at ∼1417 cm^–1^ and somewhere in between for NaSCN, which also shows an evident broadening.

The degree of deprotonation of dAA for the different salts and pH studied can be estimated from the envelope of the symmetric carboxylate stretch. In accordance with earlier studies on sodium salts at different concentrations and pH,^5,6^ the carboxylate stretch is considered to be composed of two contributing bands: a hydrated carboxylate (SIP) centered at ∼1408 cm^–1^ and a contact ion pair (CIP) between the carboxylate and the sodium counterion at ∼1417 cm^–1^. This approach is justified for the different anions, given that it is the sodium cation (and not the co-ions) that directly interacts with the negatively charged carboxylate headgroup and thus affects the peak position and amplitude. The individual contributions are obtained by fitting the spectra (eq 1) with two peaks having constrained center positions and bandwidths. The fitted amplitudes are then converted to a percentage of deprotonation by using the cross sections that have been previously estimated for both bands^5,7^ (see the Supporting Information for details of the procedure). Figure 2b summarizes the results for all salts and pH. For instance, at pH 6, having NaI in the subphase induces a ∼ 70% deprotonation, while for NaCl it is just ∼25%. Additionally, the relative proportion of the CIP increases with the degree of deprotonation of the monolayer. This is analogous to previous observations on highly charged fatty acid monolayers, where CIP formation was triggered upon reaching a critical surface charge.^5^ The carbonyl stretch at ∼1720 cm^–1^ can also be used to independently confirm the values determined from the carboxylate stretching modes. However, it is less reliable given that the orientation of the C=O bond of the uncharged acid varies with the degree of deprotonation of the monolayer^6^ (see the Supporting Information for additional details).

**Figure 3:**
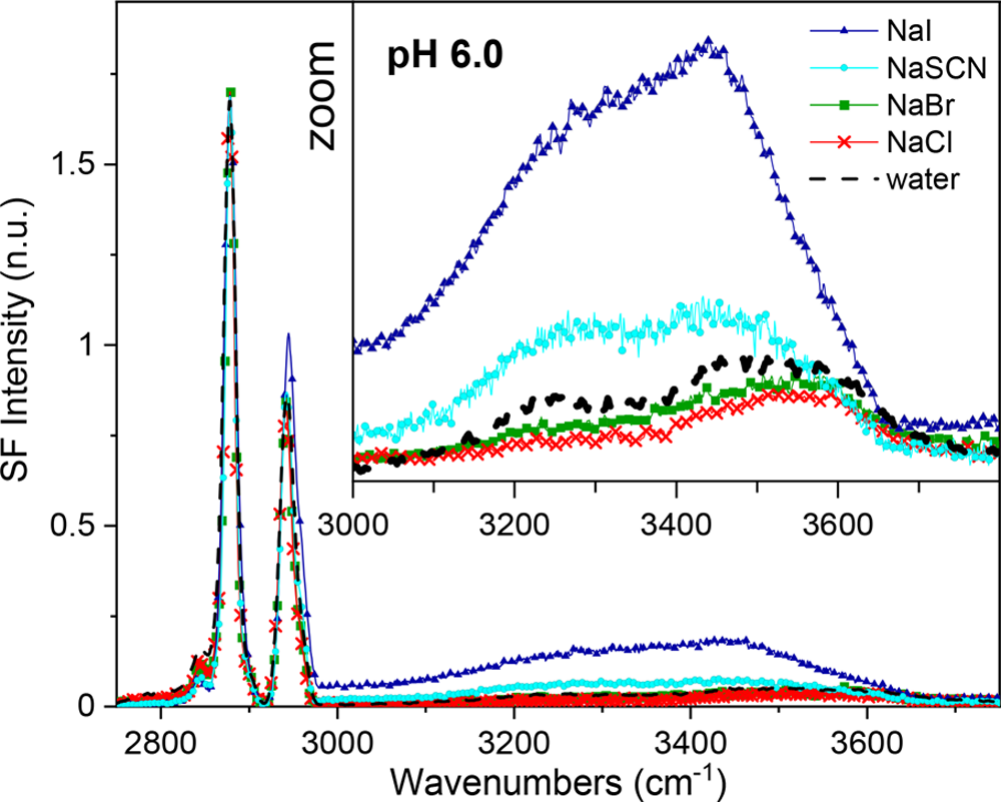
Vibrational sum frequency spectra collected under the SSP polarization in the CH and OH stretching region of an AA monolayer on water and 1 M solutions of NaCl, NaBr, NaSCN, and NaI at pH 6 and a constant surface pressure of 20 mN/m (22 °C and 20 μM EDTA). The inset shows an enlargement of the OH stretching region.

Additional insight can be obtained by targeting the response of interfacial water molecules. The VSF spectra in the OH stretching region of AA monolayers on 1 M electrolyte solutions at pH 6 are shown in Figure 3. The intensity observed between 3000 and 3650 cm^–1^ results from water molecules in direct proximity to the interface as well from those further away within the diffuse layer that are perturbed by the surface electric field.^43−45^ At concentrations of 1 M, however, the electrostatic field is substantially screened (Debye length ∼3 Å, which is the minimum value before increasing again at higher salt concentrations^46,47^), and the signal should originate from within the first 2 nm. In the case of NaCl and NaBr, the spectra look similar to that observed for a pure water subphase when the monolayer is essentially uncharged. The signal then originates primarily from water molecules directly interacting with the headgroup in its uncharged form. The two broadbands centered at ∼3275 and 3510 cm^–1^ are assigned to water molecules accepting a hydrogen bond from the fatty acid hydroxyl groups and from those donating a hydrogen bond to the carbonyl group, respectively.^48^ In contrast, the NaSCN and NaI spectra show an enhanced intensity which is consistent with their higher degree of deprotonation and corresponding surface charge. In these latter cases, the signal is mainly attributed to water molecules, not in direct contact with the headgroup, but from those partially aligned by the intense surface electric field. The SF signal from water is then consistent with the anion-specific deprotonation deduced from the headgroup vibrations, with I^–^ > SCN^–^ > Cl^–^ ≈ Br^–^. Moreover, a closer inspection of the spectra shows that the relative intensities between the contributing bands in NaSCN and NaI are different from those of water or NaCl. For NaSCN and NaI, there is an increase of the lower frequency contribution centered at ∼3200 cm^–1^, which agrees with the signal expected from water in the diffuse layer of carboxylic acid monolayers.^5−7,45^ However, in the specific case of NaI, there is an additional, relatively narrow contribution at ∼3450 cm^–1^, which indirectly indicates the presence of iodide in the surface region. The peak shares similarities to the enhanced intensity observed in the bulk Raman and liquid/vapor VSFS spectra of concentrated NaI solutions,^17,49,50^ which has been linked to water molecules in the anion hydration shell.^50^

**Figure 4:**
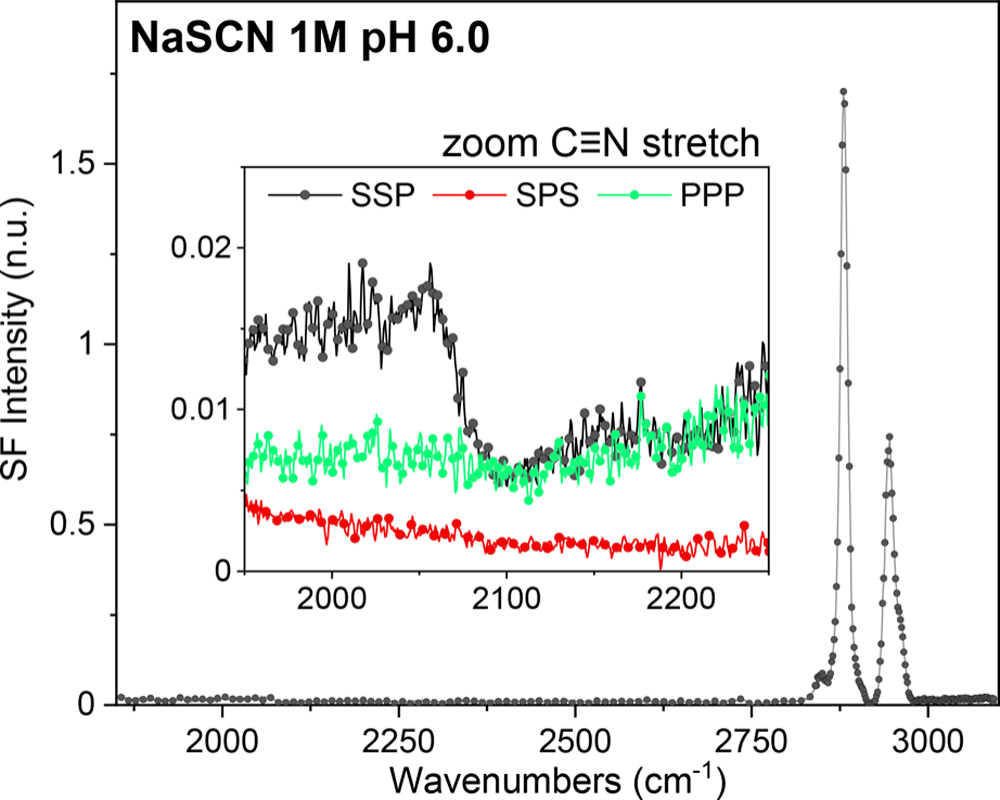
VSF spectra collected under the SSP polarization in the SCN^–^ and CH stretching region of an AA monolayer on 1 M NaSCN at pH 6 and a constant surface pressure of 20 mN/m (22 °C and 20 μM EDTA). The inset shows an enlargement of the CN stretching region, where SPS and PPP polarization spectra are also added for completion. The C≡N stretch is detected as a dispersive-shaped peak at ∼2060 cm^–1^ in the SSP spectrum.

To further explore the potential presence of co-ions in the interfacial region, we focus on the polyatomic thiocyanide anion, which can be directly targeted with VSFS.^51^Figure 4 shows the VSF spectra of an AA monolayer on a 1 M NaSCN subphase at pH 6. Although close to the detection limit, the CN triple bond of SCN^–^ can be identified at ∼2060 cm^–1^, as shown in the inset of Figure 4. The peak proves that thiocyanide anions with a preferred orientation are present in the interfacial region. However, the negligible band intensity would indicate low surface concentrations, particularly when compared to previous sum frequency studies that have investigated SCN^–^ anions at various interfaces.^51−54^

## Discussion

VSFS provides irrefutable evidence that the identity of the anion in concentrated monovalent salt solutions strongly influences the degree of deprotonation of the negatively charged carboxylic acid monolayer. In the context of electrostatic theories for charged interfaces, where co-ions are predicted to be depleted from the surface region, these results may appear striking and counterintuitive. For instance, in the Gouy–Chapman (GC) theory for surfaces with ionizable groups,^55−57^ and extensions that account for the finite ion size (MPB-steric),^58−60^ or dispersion interactions,^61,62^ only the co-ion valence (i.e., −1) is of relevance. Nonetheless, the GC model successfully predicted the charging behavior of AA monolayers at low ionic strengths (<50 mM), where the identity of the anion (and the cation) was shown not to have any influence.^7^ At higher concentrations (chloride salts up to 1 M) or highly charged surfaces (OH^–^ as co-ion), it was instead the MPB-steric model that was consistent with experimental observations, highlighting the importance of the finite monovalent cation size for limiting the accumulation of counterions at the surface.^5,6^ The significantly higher degree of deprotonation observed for the more polarizable anions, I^–^ and SCN^–^, cannot be readily accommodated by any of the two theories. For example, at pH 6, it should not exceed 40%, even when neglecting the finite size of the Na^+^ counterions (i.e., GC model).^6^

Considering potential direct anion interactions with the monolayer, numerous simulation^63−65^ and experimental^16−21^ studies have shown that large polarizable anions have a higher propensity to adsorb to hydrophobic interfaces, in the sequence SCN^–^ > I^–^ > Br^–^ > Cl^–^. In this regard, in systems where both hydrophobic and hydrophilic patches are exposed to solution (i.e., polymers and proteins), the more polarizable anions can adsorb and change the surface electrostatic properties.^15,22,66^ However, in the highly packed fatty acid Langmuir monolayers studied here, no such hydrophobic patches are exposed to solution. The anions only face the hydrophilic carboxylic acid moiety and, in particular, its negatively charged carboxylate counterpart. Although the experiments presented here show evidence for the presence of SCN^–^ and also indirectly that of I^–^ at the interface, the measured intensities for SCN^–^ were close to the detection limit, suggesting that their numbers at the interface will not be significant. The signal intensities are probably compatible with the expected relatively small enhancement of co-ions at the interface, following the layer of Na^+^ counterions packed at the steric limit, as suggested from typical atom density profiles in MD simulations.^34,67^

The next aspect to consider is the concentration of hydronium ions at the surface, which plays a central role in the headgroup’s acid/base equilibrium. The apparent p*K*_a_ of arachidic acid monolayers is ∼10.8 when only the pH determining ions are present in solution (i.e., OH^–^).^7^ That is almost 6 p*K*_a_ units higher than the intrinsic p*K*_a_ observed in the bulk (note, however, that the surface p*K*_a_ was experimentally found to be within error, equivalent to that in the bulk p*K*_a_ = 5.1 ± 0.2^7^). The significant difference between the apparent and intrinsic p*K*_a_ results from the negative surface charge and ensuing surface potential that attract hydronium ions to the surface, reaching concentrations that are several orders of magnitude higher than in the bulk. When adding salt at a constant pH, the electrostatic screening causes an absolute decrease of the surface potential (i.e., becomes less negative), and so does the surface [H^+^], leading to the partial deprotonation of the monolayer.^5−7^ In this study, the larger deprotonation observed for NaI and NaSCN solutions (Figure 2b) could then be explained by a decrease of the surface hydronium ion concentration when compared to that for NaCl or NaBr. However, the stronger SF response from water molecules in solutions with the more polarizable anions (Figure 3) indicates that the surface potential, and consequently the surface [H^+^], are not decreasing in absolute terms, but rather the opposite, as expected for a higher surface charge at a constant ionic strength. We note that spectral features linked to hydrated protons at negatively charged surfaces^13^ can be detected on these concentrated salt solutions and will be the subject of a detailed study to be presented elsewhere.

The observed behavior can, however, be accommodated by considering an anion dependence of the dissociation constant of the carboxylic acid at the surface. For all chloride salts previously investigated, the surface p*K*_a_ was experimentally found to be within error, equal to that in the bulk.^5−7^ Yet, in the presence of the more weakly hydrated anions, the data presented here show that hydronium ions can more readily unbind from the surface carboxylic acid groups (Figure 2b). The implication is that in the presence of iodide and thiocyanide anions in solution the surface p*K*_a_ is lower than the bulk value, and this by at least one p*K*_a_ unit for I^–^. The more polarizable anions would then indirectly stabilize and facilitate the charging of the monolayer. A difference between the surface and the bulk dissociation constants had been theoretically proposed, but not as a consequence of any potential co-ion effect.^68^ The behavior observed here shares similarities with that reported for the more acidic sites of negatively charged fused silica surfaces in contact with concentrated sodium halide solutions,^26^ which could also be interpreted by a decrease of the surface p*K*_a_ of silanol groups for iodide relative to chloride.^69^ Likely, the co-ion effects observed on fatty acid monolayers and fused silica surfaces have the same fundamental origin. MD simulations could be particularly useful to complement this understanding.

## Conclusions

We have shown that the degree of deprotonation of arachidic acid monolayers on concentrated aqueous solutions of monovalent salts strongly depends on the identity of the anion, with I^–^ > SCN^–^ > Cl^–^ ≈ Br^–^. The effect was confirmed in monolayers having intrinsically different charge densities (i.e., pH 6 and 9). Analysis of the SF response of interfacial water molecules indicates that the surface potential, in absolute terms, follows the same trend as the degree of deprotonation, being largest for NaI. The presence of SCN^–^ with a preferred orientation as well as I^–^ co-ions at the surface could be directly and indirectly confirmed from the SF spectra. Nonetheless, the signals detected were weak, suggesting that the anion surface concentrations are low and consistent with the relative enhancement of co-ions expected close to the surface following the layer of Na^+^ counterions packed at the steric limit. The results are incompatible with electrostatic theories for charged interfaces that only account for the ion valence (i.e., Gouy–Chapman) or its effective size (e.g., MPB). However, they can be accommodated by considering an anion dependence of the surface p*K*_a_, which is no longer the same as in the bulk in the presence of high amounts of I^–^ and SCN^–^ in solution. The underlying molecular origin of this effect remains uncertain, though dispersion forces as well as ion pairing and other subtle interactions involving the carboxylic acid moiety and the other ions in solution (i.e., H^+^, Na^+^, and I^–^ or SCN^–^) are likely at play. It is hoped that the information provided here may stimulate new theoretical and simulation studies to better understand the interactions of co-ions with hydrated protons and other counterions at negatively charged interfaces.

## Supporting Information Available

Detailed description of iodide reaction kinetics and fitting procedure for determining the proportion of contact ion pairing and degree of deprotonation (PDF)
