# Supplementary material for: Anion Specific Effects at Negatively Charged Interfaces: Influence of Cl–, Br–, I–, and SCN– on the Interactions of Na+ with the Carboxylic Acid Moiety
Source: J Phys Chem B. 2021 Oct 27;125(44):12384–91. doi: 10.1021/acs.jpcb.1c07758 (PMC8591606; doi:10.1021/acs.jpcb.1c07758)
Supplement: Supplementary file 1 — jp1c07758_si_001.pdf [file jp1c07758_si_001.pdf]

Supplementary Information

for

Anion Specific Effects at Negatively Charged Interfaces: Influence of Cl^-, Br^-, I^-, and SCN^- on the Interactions of Na^+ with the Carboxylic Acid Moiety

Adrien P. A. Sthoer, Eric C. Tyrode*

Department of Chemistry, KTH Dröttning Kristinas väg 51, SE-10044 Stockholm, Sweden

* Corresponding author: tyrode@kth.se. Telephone: +46 8 7909915

Content:

1. Chemistry of Iodide electrolytes. Page S2
2. Determination of the % of deprotonation and the relative proportion of hydrated to Na^+ paired carboxylate. Page S3

1. Chemistry of Iodide electrolytes.

In the pH range examined, the oxidized forms of iodide, mainly I_3^-, I_2, and IO_3^-, are stable in the stability range of water (green dashed lines on the Pourbaix diagram, Figure S1). The oxidation reaction of iodide with dioxygen is promoted in acidic media, following the chemical reaction: $O_{2(aq)} + 4H^+ + 6I^- \rightarrow 2H_2O + 2I_3^-$.[1-4] This reaction is spontaneous but slow in ambient conditions. The kinetic of oxidation depends on the concentration of anions, the pH, and the amount of dissolved O_2. The reaction is also accelerated when exposed to UV radiation. The presence of triiodide gives a pale yellowish tint to the solution and increases the pH by consuming H^+ ions. In order to ensure that the high deprotonation observed in the VSF spectra for NaI solutions were not due to a pH variation from iodide oxidation, 10 to 50 mM of sodium thiosulfate, a strong reducing agent, was added to the solution. The stepwise oxidation of iodide in triiodide requires an intermediate reactant, namely the iodate ion, as presented below.

$$I^- + 3H_2O \rightarrow IO_3^- + 6H^+ + 6e^-$$
$$IO_3^- + 6H^+ + 2I^- \rightarrow I_3^- + 3H_2O$$

The thiosulfate ions reduce the iodate anions, which in turn cannot further oxidize in triiodide following the reaction of the well-known iodometry titration:

$$2S_2O_3^{2-} + I_3^- \rightarrow S_4O_6^{2-} + 3I^-$$

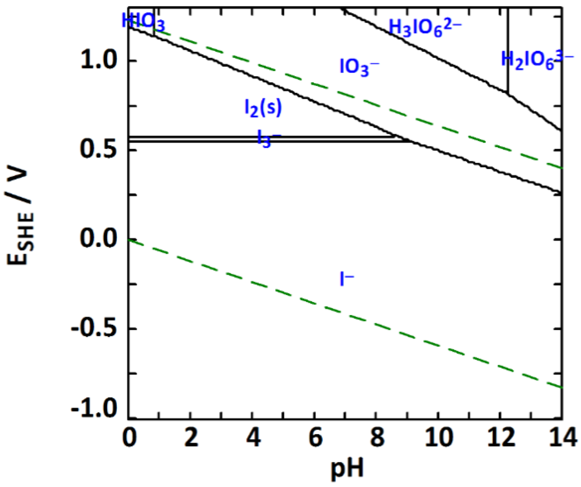

Figure S1. *Pourbaix diagram of 1M NaI aqueous electrolyte. The diagrams have been plotted using the HYDRA and MEDUSA software, with a concentration of absorbed CO_2 equal to 10^{-6} M and at 25°C.*

Values[3]: HIO_3 / IO_3^- pKa 0.75; E° (IO_3^- / $I_{2(s)}$) = 1.19 V ; E° (I_2 / I^-) = 0.62 V ; E° ($I_{2(s)}$ / I^-) = 0.53 V ; E° (I_3^- / I^-) = 0.54 V ; E° ($S_4O_6^{2-}$ / $S_2O_3^{2-}$) = 0.09.

2. Determination of the % of deprotonation and the relative proportion of hydrated to Na^+ paired carboxylate.

In order to determine the percentage of deprotonation of the monolayer, the spectra were fitted using a convolution of Lorentzian and Gaussian line shapes (see equation [1] in the article). The SF amplitude of the hydrated carboxylate allows measuring the charge of the monolayer by comparison with the fully charged monolayer on a NaOH subphase.[5] However, at high surface charge densities, the carboxylate symmetric stretch has two contributing bands,[6-7] one corresponding to the hydrated species (SIP) and the other corresponding to the Na^+-paired carboxylate (CIP), as shown in Figure S2. The fitted parameters, presented in Table S1, were constrained following a procedure described in detail elsewhere.[7] The cross-sections obtained from reference [7] were used to calculate the percentage of deprotonation of the monolayer, and the relative proportion of the two carboxylate species (Figure 2b in the article). The relative error SIP / CIP is determined by assuming the variations of amplitude within the centre position ±1 cm^{-1}. The carbonyl stretch can also be used to estimate the amount of uncharged carboxylic acid and confirm the values determined independently from the carboxylate stretching modes. However, the orientation of the C=O bond of the uncharged acid has been shown to depend on the degree of deprotonation of the monolayer,[6] making the analysis less reliable. Nonetheless, by considering the average of the $\nu_{C=O}$ intensity in both the SSP and SPS polarization combinations, the obtained % are consistent with those presented in Figure 2b.

Table S1. Constraints used for the fitting of the symmetric carboxylate band.

Vibrational mode	Center position	Lorentzian bandwidth (Γ_v)	Gaussian bandwidth (σ_v)
Hydrated COO^-	1407±1 cm^{-1}	9 cm^{-1}	4 cm^{-1}
COO^-Na^+	1417±1 cm^{-1}	9 cm^{-1}	4 cm^{-1}

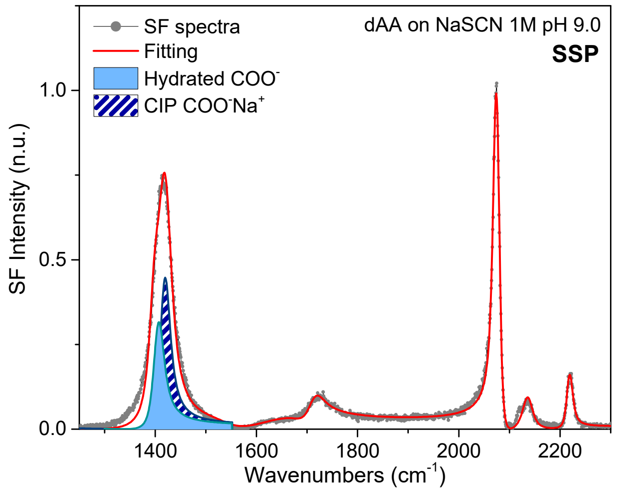

Figure S2. *VSF spectra of dAA Langmuir monolayer on NaSCN 1M pH 9.0 subphase, collected under SSP polarization, with the spectral fit shown as a red line. The light blue shaded and blue/white hatched areas represent the fitted contributions from the hydrated carboxylate, and* Na^+ *paired carboxylate modes, respectively.*

References:

1. Bray, W. C.; Liebhafsky, H. A. Reactions Involving Hydrogen Peroxyde, Iodine and Iodate Ion. I. Introduction. *Journal of the American Chemical Society* **1931,** *53* (1), 38-44.
2. Dané, L. M.; Janssen, L. J. J.; Hoogland, J. G. The Iodine/Iodide Redox Couple at a Platinum Electrode. *Electrochimica Acta* **1968,** *13* (3), 507-518.
3. Greenwood, N. N.; Earnshaw, A. *Chemistry of the Elements (Second Edition)*. Butterworth-Heinemann: Oxford, 1997.
4. Drozd, A. V.; Tishakova, T. S. Spectrophotometric Determination of Trace Amounts of Iodide-Ions in Form of Ionic Associate with Brilliant Green using Electrochemical Oxidation. *Central European Journal of Chemistry* **2011,** *9* (3), 432-436.
5. Tyrode, E.; Corkery, R. Charging of Carboxylic Acid Monolayers with Monovalent Ions at Low Ionic Strengths: Molecular Insight Revealed by Vibrational Sum Frequency Spectroscopy. *J. Phys. Chem. C* **2018,** *122* (50), 28775-28786.
6. Sthoer, A.; Hladílková, J.; Lund, M.; Tyrode, E. Molecular Insight into Carboxylic Acid–Alkali Metal Cations Interactions: Reversed Affinities and Ion-Pair Formation Revealed by Non-Linear Optics and Simulations. *Physical Chemistry Chemical Physics* **2019,** *21* (21), 11329-11344.
7. Sthoer, A.; Tyrode, E. Interactions of Na^+ Cations with a Highly Charged Fatty Acid Langmuir Monolayer: Molecular Description of the Phase Transition. *J. Phys. Chem. C* **2019,** *123*, 23037−23048.
